# Establishment of Immortalized Yak Ruminal Epithelial Cell Lines by Lentivirus-Mediated SV40T and hTERT Gene Transduction

**DOI:** 10.1155/2022/8128028

**Published:** 2022-03-25

**Authors:** JunMei Wang, Rui Hu, Zhisheng Wang, Yixin Guo, Sen Wang, Huawei Zou, Quanhui Peng, Yahui Jiang

**Affiliations:** Key Laboratory of low Carbon Culture and Safety Production in Cattle in Sichuan, Animal Nutrition Institute, Sichuan Agricultural University, Chengdu, China

## Abstract

Yak is a unique species of cattle that is adapted to the harsh natural environment of the Qinghai-Tibet Plateau. Research on the function of the yak rumen is limited to animal experiments, and the cell molecular mechanism is very limited. The high cost of isolation and culture of adult yak rumen epithelial cells (YRECs), low success rate, and limited cell life limit the scope of long-term physiological functions and nutrient absorption mechanisms of yak rumen epithelium in vitro studies. This study aimed to explore the isolation and immortal culture methods of primary YRECs and establish a new cell line model for studying cell molecular mechanisms. The human telomerase reverse transcriptase gene (hTERT) and simian virus 40 large T antigen (SV40T) were transferred into primary YDECs using mammalian gene expression lentiviral vectors. The immortalized cell line (SV40T-YREC-hTERT) retains the morphological and functional characteristics of primary cells. The epithelial cell marker protein cytokeratin 18 of the immortalized cell lines was positive, and the cell proliferation and karyotype were normal. The SV40T and hTERT genes were successfully transferred into immortalized cell lines and maintained high expression. Simultaneously, the immortalized cell lines had normal function of short-chain fatty acid (SCFA) transport and absorption, and the immortalized yak rumen epithelial cell lines were successfully established. In addition, the transepithelial electrical resistance value gradually increased with culture time, and the permeability of epithelial cells decreased by culturing epithelial cells in Transwell culture chambers. Transmission electron microscopy demonstrated the submicroscopic structure of cells in the integrity barrier model and established the YREC barrier model in vitro.

## 1. Introduction

Yak is a unique breed of cattle that is adapted to harsh environments, such as severe cold and a lack of oxygen, native to the Qinghai-Tibet Plateau. This adaptation has become an important means of life and production for local herdsmen to survive [1]. In recent years, an increasing number of herdsmen have chosen to change the rearing mode of yaks from traditional grazing to house raising and half-house raising [2]. The fattening industry of alpine yaks has gradually emerged, and rumen health is the basis for efficient yak breeding [3]. The nutrients absorbed by the rumen epithelial layer are indispensable for the energy metabolism of ruminants. Numerous epithelial papillae in the rumen extend from the surface of the rumen into the lumen, which greatly increases the surface area for absorption of volatile fatty acids and electrolytes [4]. The stratified squamous rumen epithelium is divided into the stratum corneum, granular layer, and spinous process layer and basal layer, and the basal layer and spinous process layer cells can be cultivated and grow into rumen epithelial cells [5]. In vitro cell culture can quantitatively evaluate cell morphology, structure, growth, development, and apoptosis and is widely used to study substance absorption, transport mechanisms, and energy metabolism [6, 7]. In the in vitro study of rumen development and energy metabolism, the use of isolated rumen epithelial cells has many advantages. The success rate of culturing rumen epithelial cells from neonatal animal tissues was higher, and the isolation technology for sheep was more mature [8, 9]. Earlier studies successfully isolated bovine rumen epithelial cells using trypsin stepwise digestion [10, 11]. In the process of gradually improving the trypsin concentration, type, and digestion mode, the method of culturing bovine rumen epithelial cells has made significant progress [12, 13]. Type I collagenase is commonly used to separate epithelial cells from epithelial tissues, but the combination of collagenase digestion and trypsin is more efficient and selectively removes fibroblasts to obtain epithelial cells with high purity and viability [14, 15].

Primary culture is the only way to obtain yak rumen epithelial cells (YRECs). Due to the high cost of collecting yak rumen epithelial tissue samples, the rumen is easily contaminated by microorganisms, leading to few studies on the culture of YREC at home and abroad. Notably, the limited number of tissues and primary cells has limited proliferation activity in in vitro culture, severely limiting the exploration of molecular mechanisms at the cellular level [16]. Therefore, there is an urgent need to establish stable cell lines for multiple subcultures in basic research. Current research methods for cell immortalization include simian virus 40 large T antigen (SV40T), human papillomavirus (HPV), Epstein–Barr virus (EBV), and other viruses or human telomerase reverse transcriptase (hTERT) gene transfection [17–20]. Lentivirus-infected cells induce tumor characteristics and establish immortalized cell lines [21]. Normal human cells have a finite lifespan due to replicative senescence in vitro, which is associated with progressive shortening of cell telomeres [22]. Exogenous hTERT can be successfully transferred to cells and can activate cell telomerase to lengthen telomeres, restore chromosomal stability, enable cells to pass the M1 and M2 phases, and divide and proliferate indefinitely, thereby extending the culture time or immortalization of cells in vitro [20, 23]. In recent years, studies have found that lentivirus-mediated hTERT gene transfection into cells stimulates cell proliferation, which can increase the success rate of transformation into cell lines without changing the biological characteristics of the cells [24–26]. Although stable rumen epithelial cell lines have been reported [7, 27], no research has been conducted in yak species. Therefore, the objectives of this study were to explore whether YRECs could be isolated by three different isolation and culture methods from adult yak rumen epithelium and to identify a primary culture technology for epithelial cells suitable for the characteristics of yak rumen tissues. In addition, this study established a yak rumen epithelial cell barrier model and immortalized yak rumen epithelial cell line SV40T-YREC-hTERT for the first time, providing good cell molecular biology experimental material for studying the nutritional physiology and regulatory mechanism of the yak rumen epithelial cell model in vitro.

## 2. Materials and Methods

### 2.1. Source of Rumen Tissue

Ruminal epithelial cells were isolated from the rumen epithelial tissues of adult yaks. The yak epithelium was quickly excised after death, and the tissues were placed in ice-cold phosphate buffered solution (PBS). The adult male yaks were sacrificed by Huirun Livestock Slaughter Co., Ltd. in Dujiangyan City, Sichuan Province, and the tissues were collected from the caudal sulcus of the posterior rumen abdomen.

### 2.2. Isolation and Cultivation of Primary YREC

#### 2.2.1. Type I Collagenase and Trypsin Combined Stepwise Digestion Method

The procedure for the isolation and cultivation of primary YRECs was adapted from previous studies [12, 28, 29]. The rumen nipple tissues were rinsed six times with 600 U/mL penicillin, 0.6 mg/mL streptomycin, 300 *μ*g/mL gentamicin, and 1.5 *μ*g/mL amphotericin B ice-cold phosphate buffered saline (PBS; Solarbio, Beijing, China). The rumen papilla tissues were shredded in a centrifuge tube and digested with 0.1% type I collagenase (MP Biomedicals) at 37°C for 30 minutes, and the digestive fluid supernatant was discarded. The volume ratio of enzyme to tissue was 5 : 1. Fresh 0.5% trypsin (Solarbio, Beijing, China) was added to tissues for six stepwise digestions. The supernatant was collected after each digestion, and Dulbecco's modified Eagle's medium/nutrient mixture F-12 medium (DMEM/F12; Gibco, Carlsbad, CA) containing 20% fetal bovine serum (FBS, Gibco, Carlsbad, CA) was used to terminate the enzyme digestion. Papillae were replaced with fresh trypsin digestion, and the six digestion times were 30 min, 20 min, 20 min, 15 min, 15 min, and 15 min. The digestion solution was collected by dispersing the cells in a 200 *μ*m cell sieve to collect the cell suspension, and the precipitated cells were collected in a centrifuge at 4°C for 8 min at 3000 rpm. The cell pellets were resuspended in DMEM/F12 medium containing 10% FBS, 100 U/mL penicillin, and 0.1 mg/mL streptomycin. The cells were centrifuged twice and inoculated in 25 cm^2^ culture flasks (Corning, New York, USA) coated with rat tail collagenase (Solarbio, Beijing, China) and incubated at 37°C in a 5% CO_2_ incubator. The cells were digested with 0.25% trypsin-0.02% EDTA for subculture when the cell growth density reached 90%.

#### 2.2.2. Tissue Block Culture and Tissue Block Digestion Methods

The tissue block culture method was the culture of shredded rumen papilla [30]. The tissue block digestion method was tissue block culture in the process of enzymatic digestion [31]. The tissue pieces were placed in complete cell culture medium for 5 min. The small tissue pieces were evenly spread in 10 cm petri dishes with a spacing of 5 mm. After approximately 3-4 hours in the incubator, 1 mL of complete cell medium was gently added to the upright culture flask. The entire process prevents the tissue block from floating. The complete medium was supplemented 24 h after culture, and the medium was changed every 5 days to observe the morphological changes of the cells.

### 2.3. Hematoxylin-Eosin (HE) Staining

The rumen epithelial papilla and tissue block during the enzymatic digestion process were successfully immobilized with 4% paraformaldehyde (Solarbio, Beijing, China), dehydrated, and embedded into paraffin sections. Adherent cells were fixed with 4% paraformaldehyde. According to the instructions of the hematoxylin-eosin staining kit, the staining procedures of tissues and cells were observed under an inverted microscope (Nikon, Japan) with reference to previous research methods [32], and the images were collected and analyzed.

### 2.4. Lentiviral Infection Screening and Establishment of Immortalized Cell Lines

The growth density of primary cells reached 70%. The primary YRECs were infected overnight with SV40T and hTERT lentivirus (VectorBuilder, Guangzhou, China) at a concentration of MOI = 80 and cultured at 37°C [7, 33]. Simultaneously, 5 *μ*g/mL polybrene (Sigma-Aldrich, St. Louis, MO) was used to enhance the efficiency of lentivirus infection. The medium was replaced with fresh complete medium every 2 days, and the cells were cultured for 4 days. Then, 1 *μ*g/mL puromycin (Sigma-Aldrich, St. Louis, MO) was added, and the cells were cultured for 96 h to screen cell lines. Basic information on SV40T and hTERT carriers is provided in the supplement (Figure S1). The immortalized cell lines were cultured in Roswell Park Memorial Institute (RPMI) 1640 (Gibco, Carlsbad, CA) containing 10% FBS, 10 ng/mL epidermal growth factor (EGF; R&D Systems, Minnesota, USA), 2 ng/mL basic fibroblast growth factor (bFGF; R&D Systems, Minnesota, USA), 2 ng/mL insulin-like growth factor 1 (IGF-1; R&D Systems, Minnesota, USA), 0.5 *μ*g/mL hydrocortisone (Sigma-Aldrich, St. Louis, MO), 100 U/mL penicillin, and 0.1 mg/mL streptomycin. The newly established cell lines (SV40T-YREC-hTERT) at passage 25 were sent to the China Center for Type Culture Collection (CCTCC) for preservation (CCTCC No. C2021245). The cell lines were characterized by CCTCC using species identification. The lines were free of mycoplasma.

### 2.5. Immunofluorescence Identification

The rumen epithelial tissue was successfully fixed, and tissue sections were prepared for antigen retrieval. First-generation primary cells and tenth-generation immortalized cell lines were seeded in 6-well plates, and the cell density was 50% in 4% paraformaldehyde fixative solution for 15 min. Subsequent operating procedures were performed according to the research methods in the literature [34, 35]. The cells were treated with 1% Triton X-100 in PBS for 10 min at room temperature. The tissue sections and cells were sealed with 5% goat serum (Solarbio, Beijing, China) and incubated with anti-cytokeratin 18 antibody (Abcam, Cambridge, MA, USA). The immortalized cell lines were incubated with anti-SV40T-antigen antibody (Abcam, Cambridge, MA, USA) at 4°C overnight. Protein staining was performed with fluorescein isothiocyanate- (FITC-) conjugated goat anti-mouse IgG, CY3-conjugated goat anti-mouse IgG, or FITC-conjugated goat anti-rabbit IgG secondary antibodies (Solarbio, Beijing, China) for 1 h. Each step was washed 3 times with PBS. The cell nuclei were stained with 4′,6-diamidino-2-phenylindole (DAPI; Solarbio, Beijing, China) for 5 min and then observed with a fluorescence microscope (Nikon, Japan).

### 2.6. Cell Growth Curve and Cell Viability Test

First-generation primary cells and tenth-generation immortalized cell lines were seeded in 96-well plates at a density of 5 × 10^3^ cells/mL. Six replicates were set up for each treatment group, and the vitality of one group of cells was measured every day. Cell counting kit-8 (CCK-8; AbMole, Shanghai, China) was used to measure cell viability according to the manufacturer's instructions [36]. Then, 100 *μ*L of culture medium was retained, and 10 *μ*L of CCK-8 was added to each well. The absorbance was measured at 450 nm wavelength in a microplate reader (SpectraMax M2, USA) after 4 h of incubation at 37°C. Using the incubation time as the abscissa and the average OD value as the ordinate, the cell growth curve was drawn. In an experiment to explore the influence of short-chain fatty acids (SCFAs) on cell viability, cells were seeded in 96-well plates at a density of 5 × 10^3^ cells/mL and cultured for 24 h. The cells were treated with SCFAs at different concentrations (30, 60, 90, 120, 150, and 180 mM) for 24 h. SCFAs were not added to the control group, and each group had 6 replicates. Cell viability was determined by CCK8 according to the above method. SCFAs included sodium acetate, sodium propionate, and sodium butyrate (Solarbio, Beijing, China). The 30 mM SCFA consisted of 21 mM sodium acetate, 7.5 mM sodium propionate, and 1.5 mM sodium butyrate, and the remaining concentrations were configured according to this ratio. The result calculation formula was OD value of each group/average OD of control group × 100%.

#### 2.6.1. Determination of Transepithelial Electrical Resistance

As previously described [37], 12-well polystyrene Transwell filters (1.12-cm^2^ growth area, 0.4-*μ*m pore size; Corning Costar, NY, USA) were coated with rat tail collagen. The culture chambers were placed in a 37°C incubator to equilibrate for 1 hour. The density of the first-generation cell suspension was adjusted to 1 × 10^7^ cells/mL and 1 × 10^6^ cells/mL, the density of the second-generation cell suspension was adjusted to 1 × 10^6^ cells/mL and 1 × 10^5^ cells/mL, and the cells were inoculated in the culture chamber. The integrity of the cell monolayer was confirmed by measuring TEER and paracellular permeability. Millicell®-ERS (Millipore, Billerica, MA, USA) was used to measure the resistance value. When the resistance value was increasing, the liquid was changed every day to determine the resistance value. The three wells of the culture chamber were measured separately, and the values of the three wells were averaged. No cells were inoculated in the blank group. The formula used to calculate the transmembrane resistance was TEER (*Ω* · cm^2^) = (test group resistance value‐blank resistance value) × Transwell culture chamber membrane area.

#### 2.6.2. Permeability Measurement of Transmembrane Transport Markers

The cells were washed in the culture chamber twice with PBS, the basal medium was added to the upper and lower chambers and cultured in an incubator for 30 min, 500 *μ*L dextran-fluorescein isothiocyanate (FD-4, Sigma) solution of 200 *μ*g/mL was added to the upper chamber and placed in the incubator for 1 h, and 200 *μ*L of the lower chamber mixed culture solution was added to the enzyme-labeled plate (BBI) for determination. The OD value was measured with a fluorescence microplate reader (SpectraMax M2, USA) under the conditions of excitation wavelength 485 nm and emission wavelength 582 nm. The FD4 mother liquor was diluted with basal medium to make a standard curve, and the fluorescence intensity OD value of the test sample was calculated according to the standard curve to calculate the osmotic concentration of FD4. The calculation formula of permeability coefficient is as follows: Papp = (dQ/dt)/(A × C0) [38], where dQ/dt represents the FD4 permeated during the measurement period; *A* represents the membrane area; and C0 represents the initial concentration of FD4.

#### 2.6.3. Transmission Electron Microscopy (TEM) to Evaluate the Morphological Structure of Cells after Establishing the Epithelial Barrier Model

After the transmembrane resistance and FD4 permeability were measured in the epithelial cells when they grew into a dense monolayer, the cells were washed with precooled PBS and fixed with electron microscope fixative (Solarbio, Beijing, China) for 5 min in the culture chamber. The cells were lightly shaved off the microporous membrane and collected into a centrifuge tube (placed at the bottom of the tube), which was centrifuged at high speed. The cells were fixed, dehydrated, infiltrated, embedded, sectioned, stained, and observed under a transmission electron microscope (Hitachi, Japan), and images were collected for analysis [39].

### 2.7. RNA Extraction and Quantitative Reverse Transcription Polymerase Chain Reaction (qRT–PCR) Analysis of Gene Expression

The primary cells and immortalized cell lines were verified for SCFA transport function. A total RNA extraction kit was used (Yeasen Biotechnology, Shanghai, China) to extract cellular total RNA. Gene expression was determined by qPCR as previously reported [40, 41]. The Hifair™ II 1st Strand cDNA Synthesis Super Mix for qPCR kit was used (Yeasen Biotechnology, Shanghai, China) for reverse transcription. Reverse transcription was not less than 1 *μ*g total RNA in a 20 *μ*l reaction volume. The Hieff UNICON® Universal Blue qPCR SYBR Green Master Mix kit was used (Yeasen Biotechnology, Shanghai, China) for qRT–PCR. qRT–PCR was performed using SYBR Green real-time PCR master mix in a qPCR system (QuantStudio 5, USA). RT–PCR cycles consisted of 95°C for 2 min, followed by 40 cycles of 95°C for 10 s and 60°C for 30 s. *β*-Actin was used as the internal control. The PCR primers used in the quantitative assays are listed in Table 1. The fold change in mRNA expression was determined using the 2^−ΔΔCt^ method [42].

### 2.8. Karyotype Analysis

The number of chromosomes in SV40T-YREC-hTERT was determined following standard methods [43, 44]. Briefly, the cells were treated with colcemid (Solarbio, Beijing, China) at a final concentration of 0.2 *μ*g/ml at 37°C for 5 h when they were cultured for 68 h. Cell division stopped at the intermediate phase after 2-4 h of incubation at 37°C in a 5% CO_2_ incubator. The cells were blown and mixed, transferred to a 10 ml centrifuge tube, and centrifuged at 1800 rpm for 6 minutes. The cell supernatant was removed, and the cells were treated with 8 mL of 0.075 mol/L potassium chloride at 37°C for 20 minutes. Cells were then fixed with a freshly prepared ice-cold acetic acid–methanol (1 : 3, vol/vol) solution and carefully mixed with straws before collection by centrifugation at 1800 rpm for 6 min. After, the supernatant was discarded and fixed three times in accordance with the fixed steps to make a matte suspension. The 1-2 drops of suspension are dropped onto clean slides dipped in ice water or dried, blown away, heated, and air dried. The slides were placed in a 37°C incubator for 3-4 days or baked at 70°C for 2-3 hours for aging treatment for band analysis. The chromosomes were stained with Giemsa stock (9 : 1), diluted with pH 6.8 phosphoric acid buffer for 15-20 min, washed with water, and air dried. First, the metaphase phase with good chromosome dispersion was selected under a low-power microscope, and then, the oil microscope was used for observation. Video of the TesT-Karyo chromosome karyotype analysis system for analysis is presented.

### 2.9. Data Analysis

The test results are expressed as the mean ± standard error (mean ± SEM), and statistical analysis and mapping were performed using GraphPad Prism version 5.0 software. Differences between groups were compared by one-way analysis of variance. *P* < 0.05 was considered statistically significant.

## 3. Results

### 3.1. Enzymatic Digestion Method for Isolation and Culture of Primary YREC

**Figure 1:**
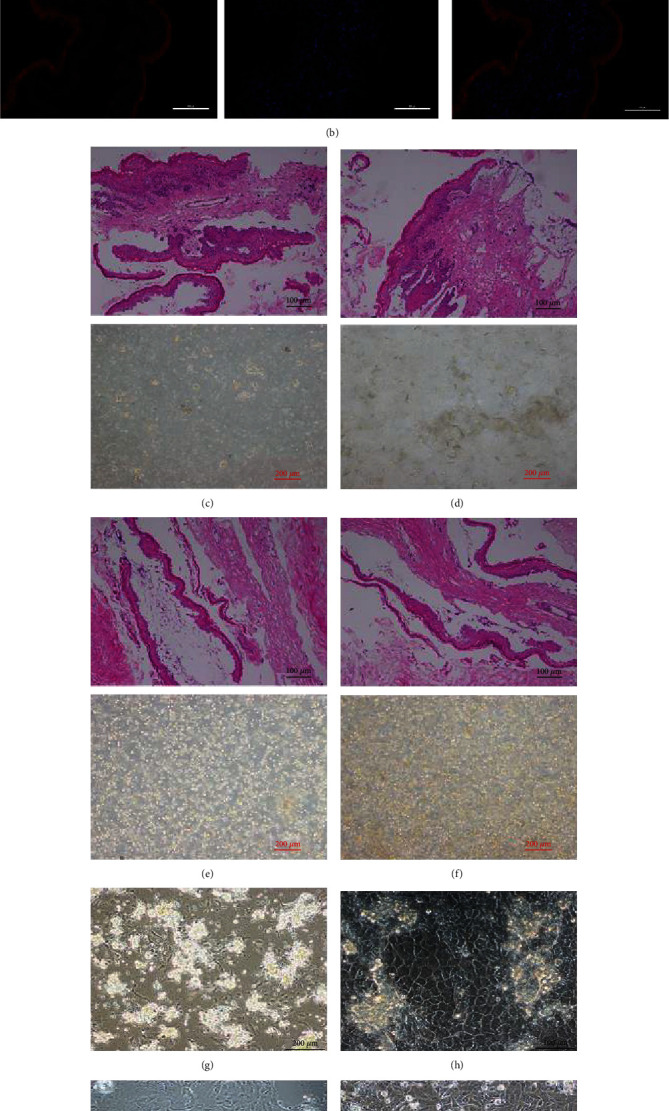
Isolation and culture of primary yak rumen epithelial cells by 0.1% type I collagenase and 0.5% trypsin combined stepwise digestion method. (a) Whole rumen epithelial tissue with HE staining (40x). (b) Immunofluorescence identification of cytokeratin 18 in rumen epithelial papilla tissue (400x). (c) The tissue structure after digestion with 0.1% type I collagenase by HE staining (200x) and the corresponding digestion fluid collected (100x). (d) The tissue structure after 0.5% trypsin digestion twice in the stepwise digestion process by HE staining (200x) and the corresponding digestion fluid collected (100x). (e) The tissue structure after 0.5% trypsin digestion four times in the stepwise digestion process by HE staining (200x) and the corresponding digestion fluid collected (100x). (f) The tissue structure after 0.5% trypsin digestion six times in the stepwise digestion process by HE staining (200x) and the corresponding digestion fluid collected (100x). (g) The collected primary yak rumen epithelial cells grew after two days (100x). (h) The collected primary yak rumen epithelial cells grew after four days (100x). (i) First-generation cell adherent growth state after primary rumen epithelial cell passage (100x). (j) The first generation of primary cells resuscitates adherent growth (100x).

Healthy and intact rumen tissues were selected, and the rumen epithelial papilla structure of the primary culture was observed by HE staining, including the stratum corneum, granular layer, spinous process layer, and basal layer (Figure 1(a)). Immunofluorescence identification showed that CK18 was specifically expressed in rumen epithelial papilla tissue (Figure 1(b)). These results indicated that many rumen epithelial cells could be collected using intact rumen epithelial tissue for experiments. HE stained the tissues selected during the entire enzymatic digestion process and showed that the stratum corneum, stratum granulosum, stratum spinosum, and stratum basale cells in the rumen epithelial tissue structure were digested. In the digestive juice, there were many highly differentiated stratum corneum cells after the first two enzyme digestions (Figure 1(c)). Epithelial cells appeared in the digestion fluid collected by trypsinization (Figures 1(d) and 1(e)). After the last two rounds of tissue digestion, many cells were collected in the digestive juice, but cells were also gradually fragmented, which was not conducive to cell adherence for growth and indicated a low survival rate. After 2 days, the cells adhered to the wall, and the cell clusters grew vigorously (Figure 1(g)). After 4 days, the rumen epithelial cells showed a compact monolayer epithelial “cobblestone paving” shape, and the cells converged to 90% confluency for subculture (Figure 1(h)). The cells maintained the same morphology as the zeroth-generation primary cells after passage for 2 days (Figure 1(i)). After resuscitation of the first generation of primary cells, the cell adherence was good, but bright vesicle-like dead cells were visible (Figure 1(j)).

### 3.2. Tissue Block Culture Method and Tissue Block Digestion Method for Culturing YRECs

**Figure 2:**
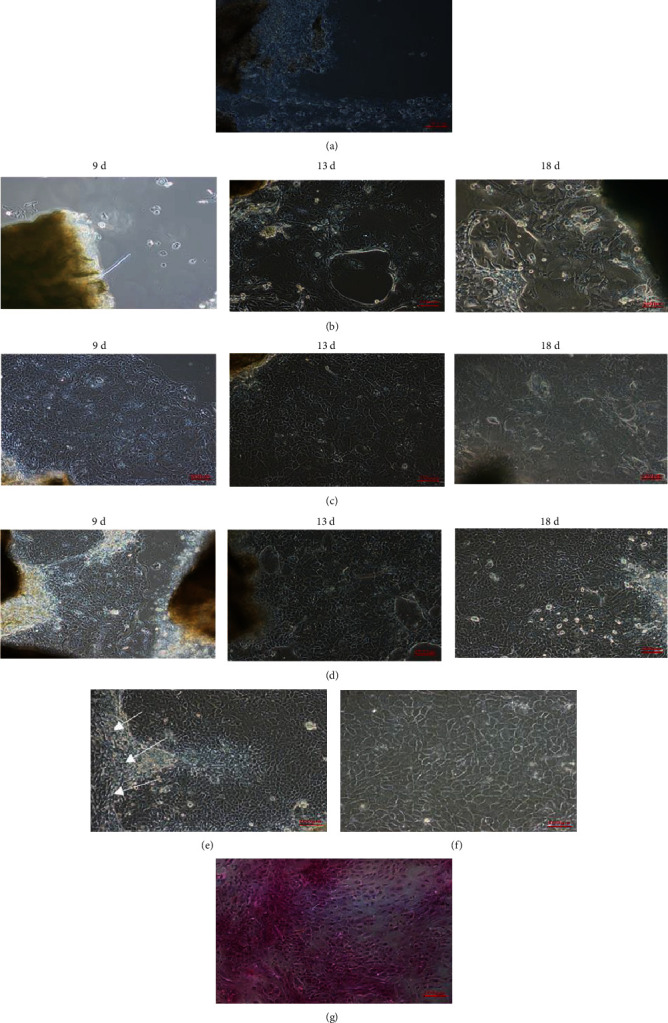
Growth status of primary yak rumen epithelial cells cultured over time using the tissue block culture method and tissue block digestion method. (a) Growth status of cells around the rumen epithelial tissue mass cultured for four days (100x). (b) Cell growth conditions around the rumen epithelial tissue mass in the tissue block culture method for 9 days, 13 days, and 18 days (100x). (c) Cell growth conditions around the rumen epithelial tissue block digested with 0.1% type I collagenase for 9 days, 13 days, and 18 days (100x). (d) Cell growth conditions around the rumen epithelial tissue block digested with 0.1% type I collagenase and 0.5% trypsin for 9 days, 13 days, and 18 days (100x). (e) Fibroblasts migrated out of the tissue mass, and rumen epithelial cells grew together (100x). (f) Morphological characteristics of typical yak rumen epithelial cells migrated out around the tissue mass (200x). (g) HE staining of yak rumen epithelial cells (200x).

The tissue blocks in the process of enzyme digestion were cultured separately, and the time and state of cell proliferation were compared between the tissue block culture method and the tissue block culture method after enzyme digestion. Four days later, no cells were found outside of the tissue blocks after the three treatments (Figure 2(a)). On the 9th day, we found that the tissue block digested with 0.1% type I collagenase and 0.5% trypsin had many cells migrate out, as shown in Figures 2(c) and 2(d). In the tissue block of the tissue block culture method, no cells were observed migrating out (Figure 2(b)). The number of cells migrating out around the tissue block gradually increased as the culture time increased, but the cell morphology gradually changed and grew into “vacuum”-like dead cells (Figure 2(b)). The morphology of the cells that migrated out by the tissue digestion culture method presented the typical morphological characteristics of epithelial cells “cobblestone paving,” forming a dense monolayer arrangement with cells around the tissue mass. Tissue block and tissue block digestion methods were prone to the phenomenon that fibroblasts migrated out first, and then, rumen epithelial cells proliferated and fused into pieces (Figure 2(e)). As shown in the figure, the primary cells obtained by the tissue block digestion method grew uniformly, and the monolayer of primary YRECs with clear and dense cell boundaries was subjected to subsequent subculture (Figure 2(f)). As shown in Figure 1, the stratum corneum and granular cells in the rumen epithelial papilla tissue structure were gradually dissociated after enzyme digestion with HE staining. The stratum spinosum and stratum basale epithelial cells were more likely to grow and proliferate without being digested by enzymes for a long time after exposure. The results of HE staining for cell passages showed that the cell boundaries were clear and polygonal, the nucleus was stained light blue–purple, the cytoplasm was stained light red, and the nucleoplasmic contrast was obvious (Figure 2(g)).

### 3.3. Screening and Establishment of Immortalized Cell Lines

**Figure 3:**
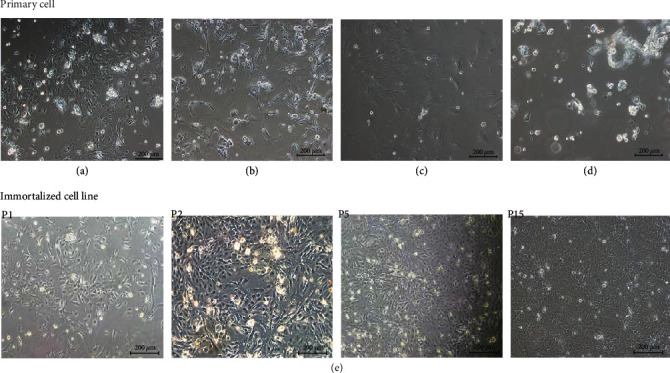
Cell state of primary yak rumen epithelial cells and established immortalized cell lines after continuous passage. (a) Cell morphology of the second-generation primary cells after adherence (100x). (b) The cytoplasm of primary cells after the third passage became larger (100x). (c) After the primary cells were passed to the third generation, the cells lost their normal epithelial cell morphology (100x). (d) After the primary cells were passed to the third generation, the cells could hardly adhere to the wall normally and gradually died (100x). (e) Simian virus 40 large T antigen (SV40T) and human telomerase reverse transcriptase (hTERT) lentivirus infection of primary cells and the cell morphology changes of the immortalized cell lines screened by puromycin at passages 1, 2, 5, and 15 (100x).

As the number of passages of primary cells increases, the cell morphology gradually changed, and cell death could not be used as a test material. For example (Figures 3(a)–3(d)), the results showed that the cells began to display larger cytoplasm after the second generation, and the irregular shape of the cells after the third generation, the cells not adhering to the wall and dying, etc., made the cells unable to be passaged and preserved. The lentivirus infected first-generation primary cells, and the cell morphology was basically the same as that of the primary cells during the screening and passage of puromycin. In the process of gradual selection and passage, the cell lines have stable morphology and can be continuously passaged. The cell adhesion rate increased, and the proliferation speed and vitality were enhanced, indicating that the lentivirus infection was successful and that the immortal cell lines were successfully established (Figure 3(e)). These cell lines have been passaged to more than 30 passages in the laboratory, and no changes in cell morphology have been found in the continuous passage. The images show the selected cells at passages 1, 2, 5, and 15.

### 3.4. Immunofluorescence Identification of Marker Proteins, Growth Curve Drawing, and SV40T and hTERT Gene Expression in Primary YRECs and Immortalized Cell Lines

**Figure 4:**
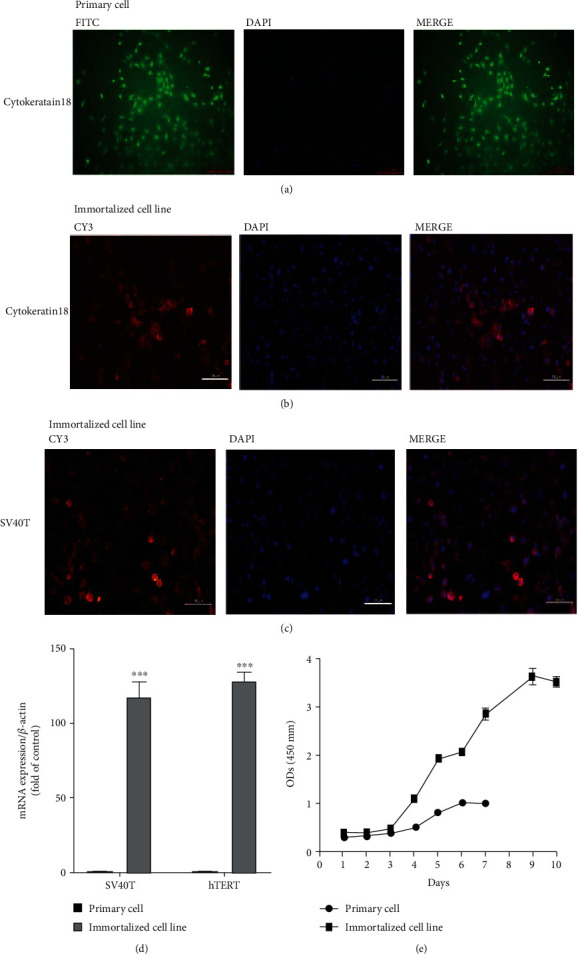
Identification and verification of first-generation primary cells and tenth-generation immortalized cell lines. (a) Immunofluorescence identification of cytokeratin 18 in primary yak rumen epithelial cells (100x). (b) Immunofluorescence identification of cytokeratin 18 in immortalized yak rumen epithelial cell lines (200x). (c) Immunofluorescence identification of simian virus 40 large T antigen (SV40T) in immortalized yak rumen epithelial cell lines (200x). (d) qRT–PCR detection of SV40T and human telomerase reverse transcriptase (hTERT) mRNA expression in primary cells and immortalized cell lines. (e) Growth curve of primary cells and immortalized cell lines.

The cytoplasm of primary YRECs and immortalized cell lines showed fluorescent expression of CK-18 protein, and the cells were epithelial-like cells by immunofluorescence detection of the epithelial cell marker CK18 (Figures 4(a) and 4(b)). When the SV40T protein was detected in the immortalized cell lines, fluorescence also appeared in the cytoplasm, indicating that the cell line was highly expressed and that SV40T was successfully transfected into the cell (Figure 4(c)). qRT–PCR results showed that immortalized cell lines stably and highly expressed SV40T and hTERT genes (Figure 4(d)). This finding further illustrated the success of the lentivirus-infected primary cells. Cell proliferation was detected by CCK8, and the growth curve is shown in Figure 4(e). The growth curve presented an “S” shape, which conformed to the growth law of epithelial cells. The proliferation ability of immortalized cell lines was significantly increased compared with that of primary cells. These results suggested that immortalized cell lines can continuously proliferate and have strong cell viability.

### 3.5. Effects of SCFA on the Transport Function of Primary YRECs and Immortalized Cell Lines

**Figure 5:**
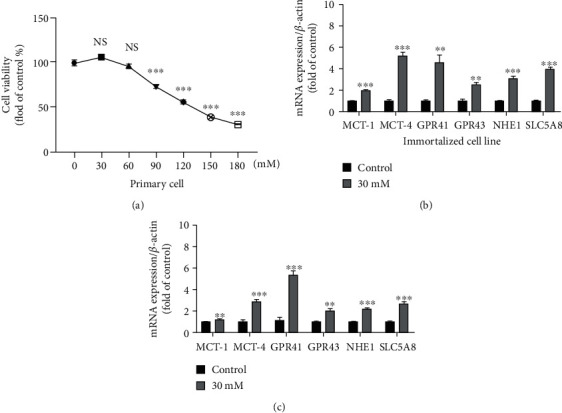
Real-time fluorescent quantitative PCR was used to verify the transfer and absorption of short-chain fatty acids (SCFAs) by primary cells and immortalized cell lines. (a) Cell viability was measured after treating the immortalized cell lines with SCFAs at different concentrations (30, 60, 90, 120, 150, and 180 mM) for 24 h. SCFAs include sodium acetate, sodium propionate, and sodium butyrate. SCFAs at a concentration of 30 mM included 21 mM sodium acetate, 7.5 mM sodium propionate, and 1.5 mM sodium butyrate. The concentrations of other SCFAs were configured according to this ratio. The data are displayed as a percentage of the control group data. (b) Effect of SCFA at a concentration of 30 mM on the mRNA expression of monocarboxylate transporter-1 (MCT-1), monocarboxylate transporter-4 (MCT-4), G protein-coupled receptor 41 (GPR41), G protein-coupled receptor 41 (GPR43), Na+/H+ exchanger isoform 1 (NHE1), and sodium-dependent monocarboxylate transporter (SCL5A8) after treatment of the first generation of primary cells for 24 h. (c) Effect of SCFA at a concentration of 30 mM on the mRNA expression of MCT-1, MCT-4, GPR41, GPR43, NHE1, and SCL5A8 after treatment of the 10th generation immortalized cell lines for 24 h. The control group was a normal growth cell group without SCFAs. Values are the mean ± SEM (*n* = 3). ^∗^, ^∗∗^, and ^∗∗∗^ indicate significant differences compared to the control at *P* < 0.05, *P* < 0.01, and *P* < 0.001, respectively. NS indicates no statistically significant difference.

We determined the cell viability of primary YRECs and immortalized cell lines at 24 h and standardized the dosage of SCFA (30, 60, 90, 120, 150, and 180 mM; Figure 5(a)). There was no significant (*P* > 0.05) difference in cell viability at 30 and 60 mM; however, SCFA significantly suppressed the cell viability at 90, 120, 150, and 180 mM compared with the control (*P* < 0.001; Figure 5(a)). The selected 30 mM SCFA concentration was used to treat the primary and immortalized cell lines to verify the transport function. Compared with the control group, SCFA induced increased mRNA expression of MCT-1, MCT-4, GPR41, GPR43, NHE1, and SLC5A8 in primary cells (Figure 5(b)). The immortalized cell lines showed the same trend and indicated that the immortalized cell lines had normal SCFA transport function (Figure 5(c)).

### 3.6. Establishment of an Epithelial Cell Barrier Model In Vitro

**Figure 6:**
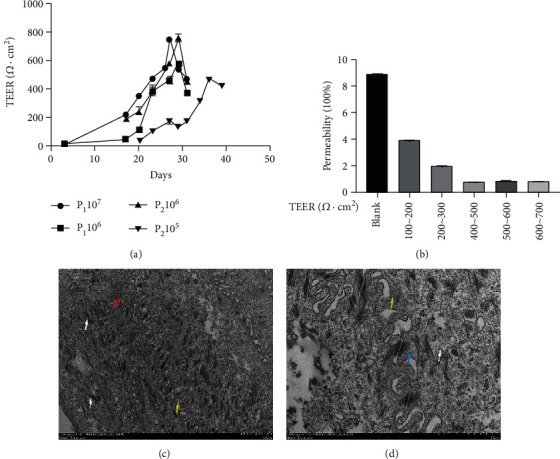
Establishment of a yak rumen epithelial cell barrier model. (a) The increasing trend of transepithelial electrical resistance (TEER) in primary yak rumen epithelial cells with different cell numbers and passage coefficients. (b) Paracellular permeability of fluorescein isothiocyanate dextran (FD4) in primary yak rumen epithelial cells with different TEER values. (c) Transmission electron microscope observation of the cell submicroscopic structure of the yak rumen epithelial cell barrier model (2500x, 10000x). Values are the mean ± SEM (*n* = 3).

There was almost no change in TEER in the first 10 days after cell seeding, indicating that the cells had not yet fused. At approximately 17 days, the cells with the highest seeding density of the first and second generations began to converge and grow into a monolayer, and the TEER value changed significantly. At approximately 20 days, the TEER value changed significantly, and cell growth was slow when the seeding density was small. The highest TEER value reached 759.36 ± 25.94 *Ω* · cm^2^ (Figure 6(a)). The results show that the primary YREC can form a complete and dense cell monolayer with increasing culture time, meeting the requirements of the experiment. By detecting the transmittance of the fluorescent marker FD-4 with the TEER value at different stages, the results show that the transmittance of FD-4 was relatively high when the TEER value was below 200 *Ω*·cm^2^, and a compact and complete cell monolayer was not formed at this time. As the TEER value gradually increases, the transmittance of FD-4 gradually decreases, and when the TEER value reached 400 *Ω*·cm^2^ or more, the transmittance of FD-4 tends to be a stable value, and the permeability is below 1% (Figure 6(b)). The results showed that the epithelial cell monolayer had good integrity and met the test requirements. Further observation of the submicroscopic structure of the cells after the cells grew into a compact monolayer showed that the cell structure was intact, and there was a tight junction structure at the outer top of the cell membrane. There were microvilli protrusions between the cells, arranged tightly and neatly, as shown by the yellow arrows, and mitochondria (red arrows) and bundles of tension microfilaments (white arrows) were seen in the cytoplasm (Figure 6(c)). The microvilli protrusions are shown by the yellow arrows. The microvilli between the cells were often connected by desmosomes, as shown by the blue arrows, and tension microfilaments in bundles were seen, as shown by the white arrows (Figure 6(d)).

### 3.7. Stable Chromosomes of the Immortalized Cell Line SV40T-YREC-hTERT

**Figure 7:**
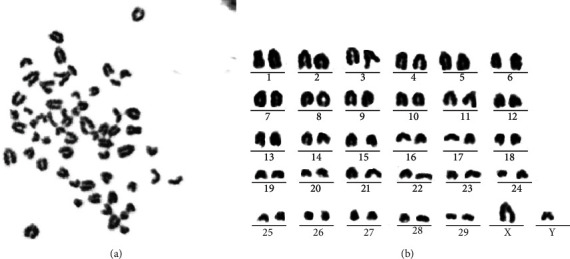
Karyotype analysis of immortalized yak rumen epithelial cell lines (SV40T-YREC-hTERT). (a) Karyotype analysis of SV40T-YREC-hTERT cells. (b) Chromosomal rearrangements of SV40T-YREC-hTERT.

Karyotype analysis was performed to determine the karyotype stability of the immortalized cell line SV40T-YREC-hTERT. As shown in Figure 7(a), SV40T-YREC-hTERT showed typical and representative diploid characteristics of yak species (2*n* = 60) without mutation to polyploidy. Chromosome rearrangement is shown in Figure 7(b), including 29 autosomal pairs and one sex chromosome pair.

## 4. Discussion

As an important organ for ruminant digestion, metabolism, nutrient absorption, and the immune barrier, the rumen has been increasingly studied domestically and internationally, and the regulatory mechanism in rumen epithelial cells has also attracted much attention. The yak is a dominant species that lives in a special geographical environment year round, and its rumen metabolism has a certain specificity [45]. To explore the experimental research functions of the YREC in vitro culture environment more conveniently and deeply, such as growth, differentiation, metabolism, pathological changes, drug intervention, and other experimental research functions, it is very important to establish a suitable YREC culture method and stable immortalized cell lines.

The most common methods for tissue isolation and primary culture of epithelial cells are enzyme digestion and tissue mass culture. Enzymatic digestion methods include trypsin, collagenase, and multiple enzyme-combined digestion [15]. Collagenase can specifically decompose fibrous collagen in the intercellular substance with less destructiveness and is mostly used for digestion and separation of fibrous, epithelial, and other tissues [46]. Trypsin mainly acts on the peptide bond connected by lysine or arginine to hydrolyze the protein in the intercellular substance, and the digestion time has a greater impact on the cell [47]. The isolation and culture methods of primary cells are different for different animal species, ages, and individuals. In this study, the isolation and culture methods of rumen epithelial cells and epithelial cells in different parts of different animals were used for reference, and the success rate of single trypsin digestion was lower, and fewer cells were isolated [6, 48, 49]. To further improve the enzyme concentration, digestion steps, and digestion time, 0.1% type I collagenase digestion was selected for 30 minutes, followed by 0.5% trypsin 6 times. Undifferentiated rumen epithelial cells with proliferative activity mainly exist in the spinous and basal layers of epithelial tissues [37, 50]. HE-stained digestive tissues showed that the stratum corneum, granular layer, spinous process layer, and basal layer gradually fell off as the number of digestions increased during the digestion process. The presentation of the entire digestion process has guiding significance for the separation and culture method. If the trypsinization time is too long, the cells are almost fragmented and unable to grow adherently, which is extremely harmful to the cells. The digestion time of the enzyme digestion method must be strictly controlled to reduce the impact on cell viability and increase the success rate.

Compared with the enzymatic digestion method, the tissue block culture method is simple and easy to perform, and primary cells in a state closer to tissue cells can be obtained [51]. The tissue block culture method has a long cycle, and the enzymatic digestion method causes greater cell damage. Combined with the enzyme digestion method and tissue culture method to isolate primary cells, the culture time can be shortened, and a large number of stable cells can be obtained [52–54]. Type I collagenase digests tissue blocks for 30 minutes to separate cells from the collagen components without affecting the cells themselves, ensuring cell viability and easy cell separation. In this experiment, the tissue block digested by the combination of collagenase and trypsin was cultured, while the tissue block digested with trypsin was not cultured. On the one hand, this method prevents excessive digestion and damage to cells that do not easily migrate. On the other hand, the increase in digestion time will increase the viscosity of the tissue after enzymolysis and make it difficult to adhere [55]. In this experiment, the tissue block culture after the combined digestion of collagenase and trypsin produced more epithelial cells with consistent morphology in a shorter time than the tissue block culture. The cells grown by the three tissue culture methods had fibroblasts, which were removed by the differential adhesion method and trypsin digestion, and the epithelial cells were purified to obtain high-purity primary rumen epithelial cells [6, 8, 56].

The number and culture time of primary cells were limited each time. When rumen epithelial cells passed to the third generation, they could not proliferate, and their lifespan was limited by cell senescence. In addition, due to the different physiological states of repeatedly separated cells, there may be differences between different batches of cells. The SV40T or hTERT gene was introduced into primary cells to increase the immortalization probability and obtain immortalized cell lines with immortal proliferation ability [57, 58]. Viral gene expression can lead to changes in cell proliferation and various “transformed” phenotypes associated with tumors. The SV40 sequence can be used in various ways, where replicative cell senescence can be prevented using defective structures of human cell origin or a single cloned cDNA [59]. SV40T is required for immortalization, but the efficiency of a single use of this gene is not high. One study found that cells may restore the life cycle of uninfected cells when the culture material or environment changes [60]. In the expected use of this experiment, SV40T alone was also found to have a low success rate and transfection of nontarget positive cells. Previous studies have shown that the main cause of replicative senescent cells is related to the shortening of telomerase [61]. Exogenously introduced hTERT gene overexpression in cells prevents telomeres from shortening, initiates telomerase activation, allows cells to gain the ability to proliferate indefinitely, and maintains normal physiological and biochemical characteristics [58, 62]. In this study, lentiviral transduction of SV40T and hTERT genes was used to prevent cell senescence and improve the conversion rate of immortalized phenotypes. The SV40T and hTERT genes were stably and highly expressed in passaged immortalized cell lines, and immortalized yak rumen epithelial cell lines with the morphology and function of the primary cells were successfully obtained. Appropriate medium is essential to maintain healthy cell growth. The addition of exogenous growth stimulating factors, including EGF, basic fibroblast growth factor (FGF), insulin-like growth factor 1 (IGF-1), and insulin, can promote epithelial cell proliferation and cell cycle progression [63, 64]. After the medium composition was optimized, the cell growth rate and passage coefficient were improved.

Cytokeratin 18 is a member of the intermediate filament gene family of epithelial cells and an important protein marker for identifying epithelial cells [65]. The rumen epithelial barrier prevents bacteria, toxins, and other harmful substances from invading the body. Its structural basis is complete rumen epithelial cells and tight junctions between epithelial cells [66]. The growth and fusion of rumen epithelial cells in the culture chamber produced higher TEER values, indicating that the normal expression of functional tight junction proteins can establish the rumen epithelial cell barrier model. The TEER value of epithelial cells reached a plateau value after 30 days of culture time, and the highest TEER value reached 700 *Ω* cm^2^, which exceeded the TEER value of 300-500 *Ω* cm^2^ of intact epithelial cells [37, 67]. Epithelial permeability is an important indicator for testing the barrier function of epithelial cells. The higher the transepithelial resistance is, the lower the cell permeability and the better the integrity of the barrier function [68]. The ratio of fluorescein-labeled macromolecules passing through the epithelial barrier reflects cell permeability. The permeability at different stages of resistance rise was investigated experimentally. The permeability of dextran was observed to be less than 1%, which was consistent with previous studies on the permeability of calf foregastric epithelium [38]. Alternatively, the morphological observation of rumen epithelial cells can more accurately reflect the ultrastructure changes of epithelial barrier cells [69]. The barrier model established by rumen epithelial cells had a complete structure and normal organelles.

SCFAs are the main energy source for ruminants, providing up to 80% of rumen maintenance energy requirements, mainly acetate, propionate, and butyrate [70]. Various studies have shown that monocarboxylic acid transporter and SLC5A8 directly or indirectly participate in the intracellular transmembrane transport of SCFA and its metabolites into the blood [71]. SCFAs induce the expression of NHE1 in epithelial cells and increase activity through diffusion and absorption of hydrogen or sodium ions to maintain pH homeostasis in the rumen [72]. Previous studies have shown that GPR43 and 41 are SCFA receptors that regulate host energy metabolism and the immune response [73]. In this experiment, SCFA induced the expression of MCT-1, MCT-4, GPR41, GPR43, NHE1, and SLC5A8 in primary rumen epithelial cells and immortalized cell lines, indicating that the immortalized cell lines had physiological and biochemical functions of rumen epithelial cells. In previous studies, bovine ruminal epithelial cell lines successfully induced by SV40T did not have GPR43 receptor gene expression [7]. The yak rumen epithelial cell lines induced by SV40T and hTERT successfully expressed the GPR43 receptor gene, providing an ideal immortalized cell line for future experimental research.

## 5. Conclusion

In summary, this experiment cultured adult yak primary rumen epithelial cells by exploring different isolation and culture methods of enzyme digestion, tissue block culture, and tissue block digestion and culture and tracked and compared the entire isolation and culture process. The YREC barrier model was established in vitro, and the cells were infected with lentiviruses carrying the SV40T and hTERT genes. Positive cells were screened out by puromycin, and the appropriate culture method was explored. The cell growth rule was determined to verify the transfer and absorption function of the immortalized cell lines for SCFA, and the immortalized cell line SV40T-YREC-hTERT was established for the first time. SV40T-YREC-hTERT provided an experimental model for studying the biological function of the yak rumen epithelium in vitro.

## Figures and Tables

**Table 1:** Primers used for real-time PCR.

Gene	Forward (5′→3′)	Reverse (5′→3′)
SV40T	AATTTGCCCTTGGACAGGCT	GCCTGAAATGAGCCTTGGGA
hTERT	GTATGCCGTGGTCCAGAAGG	CGTGGGTGAGGTGAGGTGTC
GPR41	TCGACCCCCTTGTCTACTATTTCTC	CCCAGCAATCCGTGGAAGT
GPR43	GACCCCTTGCTTTTCTATTTCTCTTC	TGCCTGCAGCCCTTTCC
MCT-1	TATGGTGGAGGTCCTATCAGCAGTG	GTGTTACAGAAGGAAGCAGCAATCAAG
MCT-4	CATGGTGTCTGCGTCCTTCTGTG	AAGTAGCGGTTGAGCATGATGAGTG
NHE-1	AGCCGCTCTTCGTCTTCCTCTAC	GTGTGGGACTTGTGGGAGATGTTG
SLC5A8	ACTGGAGGACACTGTGCTTAATGTTC	GCTCATGCCTTACTTGGTACTGGAC
*β*-Actin	TCACGGAGCGTGGCTACAG	TTGATGTCACGGACGATTTCC

## Data Availability

The data used to support the findings of this study are available from the corresponding author upon request.

## Supplementary Materials

Supplementary Materials Figure S1: basic information of simian virus 40 large T antigen (SV40T) and human telomerase reverse transcriptase (hTERT). (a) SV40T vector map. (b) hTERT vector map.
