# Supplementary material for: Establishment of Immortalized Yak Ruminal Epithelial Cell Lines by Lentivirus-Mediated SV40T and hTERT Gene Transduction
Source: Oxid Med Cell Longev. 2022 Mar 25;2022:8128028. doi: 10.1155/2022/8128028 (PMC8975702; doi:10.1155/2022/8128028)
Supplement: Supplementary Materials — Figure S1: basic information of simian virus 40 large T antigen (SV40T) and human telomerase reverse transcriptase (hTERT). (a) SV40T vector map. (b) hTERT vector map. [file 8128028.f1.docx]

**Supporting Information for**

**Establishment of Immortalized Yak Rumen Epithelial Cell Lines by Lentivirus-Mediated SV40T and hTERT gene Transduction**

JunMei Wang^1＃^, Rui Hu^1＃^, Zhisheng Wang^1^*, Yixin Guo^1^, Sen wang^1^, Huawei Zou^1^, Quanhui Peng^1^ and Yahui Jiang^1^

^1^Key Laboratory of Low Carbon Culture and Safety Production in Cattle in Sichuan, Animal Nutrition Institute, Sichuan Agricultural University, Chengdu, China.

* Corresponding Author: Zhisheng Wang, Low Carbon Breeding Cattle and Safety Production University Key Laboratory of Sichuan Province, Animal Nutrition Institute, Sichuan Agricultural

University, Chengdu 611130, Sichuan, China. E-mail: [zswangsicau@126.com](mailto:zswangsicau@126.com)

＃These authors contributed equally to this article.

**
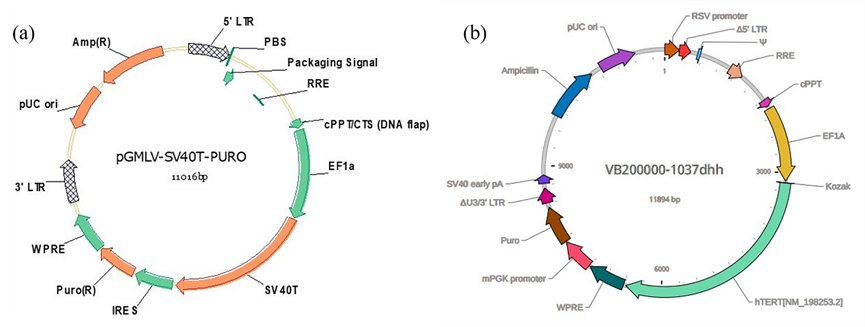
**

**Figure S1. Basic information of simian virus 40 large T antigen (SV40T) and human telomerase reverse transcriptase (hTERT).** **(a) SV40T** **vector map. (b) hTERT vector map.**
